# Pembrolizumab Therapy Leading to Complete Remission for Recurrence of Pulmonary Metastases after their Resection and Radical Cystectomy following Gemcitabine and Cisplatin Therapy

**DOI:** 10.1155/2024/5586448

**Published:** 2024-01-17

**Authors:** Kyohei Ishida, Go Hasegawa, Takehisa Hashimoto, Yohei Ikeda, Noboru Hara, Tsutomu Nishiyama

**Affiliations:** ^1^Department of Urology, Uonuma Institute of Community Medicine, Niigata University Medical and Dental Hospital, Minamiuonuma, Niigata, Japan; ^2^Department of Pathology, Uonuma Institute of Community Medicine, Niigata University Medical and Dental Hospital, Minamiuonuma, Niigata, Japan; ^3^Department of Respiratory Surgery, Uonuma Institute of Community Medicine, Niigata University Medical and Dental Hospital, Minamiuonuma, Niigata, Japan; ^4^Department of Diagnostic Radiology, Uonuma Institute of Community Medicine, Niigata University Medical and Dental Hospital, Minamiuonuma, Niigata, Japan

## Abstract

A 64-year-old man was diagnosed with invasive bladder and right lower ureteral urothelial cancer with right pelvic lymph node and lung metastases. He received four courses of gemcitabine and cisplatin therapy. He underwent lung metastasectomy and radical cystoprostatectomy, with not only primary lesions but also metastatic lesions showing a complete response. New multiple lung metastases were revealed five months after adjuvant chemotherapy. On starting pembrolizumab therapy, the metastatic lesions are notably reduced in size. He is currently receiving pembrolizumab therapy, and no recurrence has been observed for over one year.

## 1. Introduction

Chemotherapy, such as gemcitabine and cisplatin (GC), or the use of immune checkpoint inhibitors (ICIs), such as pembrolizumab, for metastatic urothelial carcinoma rarely leads to durable remission [1, 2]. We encountered a patient who received pembrolizumab therapy, leading to complete remission for recurrence of pulmonary metastases after their resection and radical cystectomy following GC therapy for invasive bladder and right lower ureteral cancer with pelvic lymph node and lung metastases.

## 2. Case Presentation

**Figure 1:**
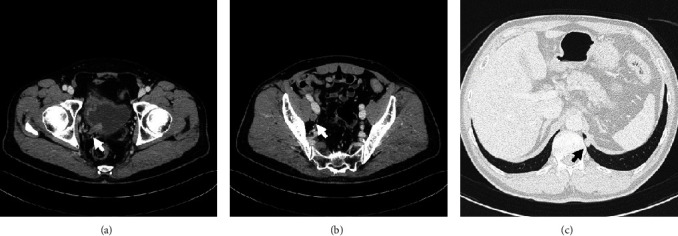
CT in April 2020 showed tumors extending from the right bladder wall to the right lower ureter (a) (arrow) with right pelvic lymph node metastasis (b) (arrow) and lung metastases (c) (arrow).

**Figure 2:**
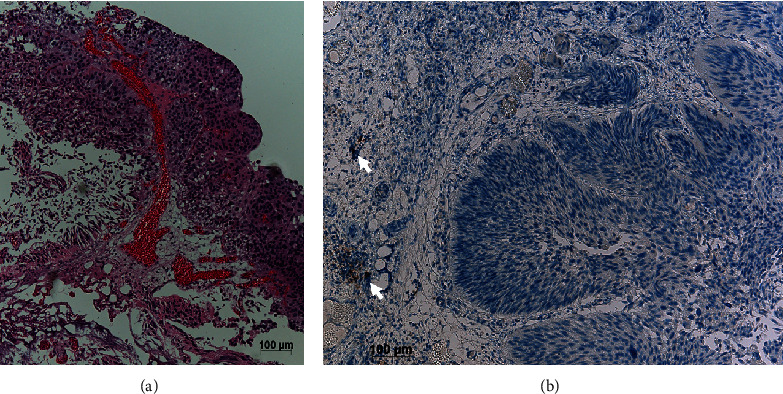
Pathological findings following transurethral resection of bladder tumors reveal invasive urothelial carcinoma, G2 high grade (a) and PD-L1 negative (b). Internal control macrophages are positive (arrow).

**Figure 3:**
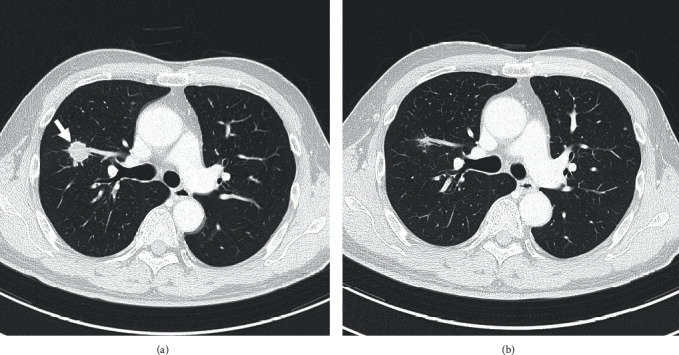
(a) CT in May 2022 showed new multiple lung metastases (arrow). (b) CT in July 2022 showed that the metastatic lesions had notably reduced in size, and no new lesion was detected.

A 64-year-old man visited our hospital complaining of gross hematuria in April 2020. Cystoscopy showed multiple tumors centering around the right ureteral orifice. Computed tomography (CT) showed tumors extending from the right bladder wall to the right lower ureter with right pelvic lymph node and lung metastases (Figure 1). Pathological findings obtained following transurethral resection of bladder tumors revealed invasive urothelial carcinoma, G2 high grade (Figure 2). He was subsequently diagnosed with invasive bladder and right lower ureteral urothelial cancer with right pelvic lymph node and lung metastases. The patient initially received GC therapy. After completing two courses of GC therapy, both the primary and metastatic lesions were reduced, so we examined the subsequent treatment policy with a cancer board. In the meeting, the participants studied the present case and discussed the treatment strategy. After 4 courses of GC therapy, we planned to resect lung metastases, and, if there was no residual cancer, we planned to perform radical cystectomy. He received four courses of GC therapy as planned. CT after the chemotherapy showed that the primary and metastatic lesions had notably reduced in size. Thoracoscopic pulmonary metastasectomy in August 2020 revealed no residual cancer tissue. He underwent radical cystoprostatectomy with right lower ureter and pelvic lymphadenectomy, and neobladder construction using the ileum in September 2020. Pathological findings revealed no remnant of malignant tissue in the bladder or right lower ureter and lymph nodes. We proposed the patient about the treatment options: adjuvant immune checkpoint inhibitor therapy, adjuvant platinum-based chemotherapy, or active surveillance. The patient hoped for adjuvant platinum-based chemotherapy. He received adjuvant chemotherapy involving two courses of gemcitabine and carboplatin instead of cisplatin because of a lower renal function [3]. Follow-up CT in May 2022 showed new multiple lung metastases (Figure 3(a)). He began to receive pembrolizumab therapy from May 2022 (200 mg once every 3 weeks). CT in July 2022 showed that the metastatic lesions had notably reduced in size, and no new lesion was detected (Figure 3(b)). The patient developed hypopituitarism and consequently has been receiving supplemental glucocorticoid treatment since July 2023. He is currently receiving pembrolizumab therapy (400 mg once every 6 weeks from October 2022), with no recurrence observed as of October 2023.

## 3. Discussion

Metastatic urothelial cancer generally remains an incurable condition [2]. Systemic therapy is the primary treatment for muscle-invasive bladder cancer (MIBC) and metastatic urothelial cancer, primarily involving platinum-based chemotherapy. The most frequently employed regimens are GC and methotrexate, vinblastine, doxorubicin, and cisplatin (MVAC). Gemcitabine plus carboplatin, a less effective alternative to GC for metastatic urothelial cancer, has been examined in retrospective studies involving cisplatin-ineligible patients with MIBC [3]. Recently, in the VESPER trial, dose-dense MVAC was reported to improve progression-free survival [4]. In the present case, we confirmed a complete response for not only primary lesions but also metastatic lesions following four courses of GC therapy. Petrelli et al. reported that patients with bladder cancer who achieved a complete pathological response after neoadjuvant chemotherapy show more favorable overall and progression-free survival rates than patients without a complete pathological response [5]. Some patients were reported to benefit from immediate chemotherapy regarding overall survival after radical cystectomy and bilateral lymphadenectomy for muscle-invasive urothelial carcinoma [6]. Following lung metastasectomy and cystoprostatectomy, we treated the present patient with adjuvant chemotherapy involving two courses of gemcitabine and carboplatin instead of cisplatin because of a lower renal function. However, he relapsed five months after treatment.

Since the relapse, we have been treating the patient with pembrolizumab therapy. The use of ICIs such as pembrolizumab has improved the survival outcomes of patients with metastatic urothelial cancer [1]. Pembrolizumab led to significantly longer overall survival (approximately 3 months) than chemotherapy in patients with previously treated metastatic urothelial cancer; however, achieving complete remission is rare. First-line pembrolizumab was reported regarding its potential to improve the prognosis of patients with early relapsing disease after receiving platinum-based perioperative chemotherapy [7]. The present patient has been receiving pembrolizumab therapy for over one year, achieving complete remission.

Recently, it was reported that there may be an association between the efficacy of chemotherapy or radiotherapy and effectiveness of subsequent ICI treatment [8]. We have also encountered patients for whom chemotherapy and radiotherapy were effective, including the present case, but subsequent pembrolizumab therapy led to complete remission [9, 10]. In the present patient, various scenarios can be considered retrospectively. The patient could have avoided radical cystectomy by receiving ICI therapy following GC chemotherapy. The JAVELIN Bladder 100 trial demonstrated significantly longer overall survival with first-line maintenance avelumab versus best supportive care in patients with metastatic urothelial cancer who achieved at least stable disease with a minimum of 4 lines of first-line platinum-based chemotherapy [11]. Furthermore, the present patient could have avoided recurrence with adjuvant ICI therapy following radical surgery. In the phase 3 CheckMate 274 trial involving patients with high-risk muscle-invasive urothelial carcinoma who had undergone radical surgery, disease-free survival was longer with adjuvant nivolumab than placebo in an intention-to-treat population and among patients with a PD-L1 expression level of 1% or more [12]. The cancer tissue in the present patient did not express PD-L1; however, complete remission was achieved with pembrolizumab therapy.

The biomarkers for predicting therapeutic efficacy remain to be identified [13]. Recently, the present patient developed hypopituitarism and has been receiving supplemental glucocorticoid treatment. One potential clinical biomarker for the ICI response in patients is immune-related adverse event (IrAE) onset [14, 15]. A growing number of studies suggest that IrAE onset is predictive of the ICI response across a variety of solid tumors. IrAEs may represent a clinical biomarker of the ICI response. The identification of biomarkers that could help to predict therapeutic efficacy is of marked clinical importance for determining the treatment outcome.

## 4. Conclusion

We described a patient who received pembrolizumab therapy, leading to complete remission for recurrence of pulmonary metastases after their resection and radical cystectomy following GC therapy for invasive bladder and right lower ureteral cancer with pelvic lymph node and lung metastases. With advances in treatment methods, the number of patients with metastatic urothelial cancer achieving a cure will increase, as in the present case.
